# Anthropometric Breast Measurement: Analysis of the Average Breast in Young Nulliparous Saudi Female Population

**DOI:** 10.1097/GOX.0000000000002326

**Published:** 2019-08-08

**Authors:** Mohammad M. Al-Qattan, Sahar S. Aldakhil, Turki S. Al-Hassan, Abdulah Al-Qahtani

**Affiliations:** From the * College of Medicine, King Saud University, Riyadh, Saudi Arabia; † College of Medicine, King Saud bin Abdulaziz University for Health Sciences, Riyadh, Kingdom of Saudi Arabia; ‡ Department of Plastic Surgery, National Guard Hospital, Riyadh, Saudi Arabia; § Department of Plastic Surgery, National Guard Hospital, Riyadh, Kingdom of Saudi Arabia.

## Abstract

Anthropometric measurements and proportions of the female body play a significant role in plastic and reconstructive surgery. This study is aimed to identify the descriptive measurements of the breast in a sample population of young nulliparous Saudi women.

**Methods:**

Fifty-four nulliparous Saudi women of 20–25 years old, with no physical or developmental deformity, and body mass index (BMI) of 20–25 kg/m^2^ were recruited. The following parameters were measured: body weight, height, BMI, sternal notch-nipple length for each breast (SN), internipple distance (IND), distance from nipple to inframammary fold (IMF), distance from edge of areola to the IMF, and areolar diameter (AD).

**Results:**

The mean values of age, BMI, height, and weight of the participants were 22.1 ± 1.2 years, 21.8 ± 3.1 kg/m^2^, 162.1 ± 5 cm, and 57.4 ± 8.6 kg, respectively. The mean values of the breast parameters were 19.8, 20.3, 7.7, 5.4, and 4.5 cm for SN, IND, distance from nipple to IMF, distance from lower end of the areola to IMF, and AD, respectively. Statistically significant difference was found only in the distance from edge of areola to IMF between the right and left breasts, with the parameter significantly higher in the left breast (*P* < 0.05, n = 54). A positive correlation between weight and BMI was found in SN, IND, distance from nipple to IMF, and AD.

**Conclusion:**

These study results will be useful for the comparison of anthropometric breast values of young Saudi women with those of women from other nationalities.

## INTRODUCTION

Plastic surgical procedures are typically planned to meet patient demand and satisfaction.^1^,^2^ However, the surgeon’s role and opinion before any procedure are considered crucial as aesthetic surgeries such as breast surgery are viewed as an exercise in artistic skill rather than science.^3^ Nonetheless, understanding the aesthetic ideals of the body is fundamental to aesthetic surgery.

The female breasts are important and attractive features, and their shape and size are subject to numerous factors. In addition to the several anatomical variations of breasts such as length, projection, width, and placement on the chest wall, breast appearance can also be affected by hormonal factors associated with puberty, pregnancy, lactation, and menopause. Furthermore, factors such as age, heredity, physical activity, and congenital diseases also affect breast dimensions.^4,5^ The effects of these variable factors and the results due to posture make precise reproducible measurements of breast morphology, which require remarkable skill, a difficult task.^6^

Objective and standardized measurements are absolutely necessary; however, there are limited acceptable systems of measurement. Several methods have been described in relevant literature, from 1- to 3-dimensional imaging, to evaluate breast shape, projection, volume, or upper pole fullness.^6–8^ One of the observations in literature is the differences in breast anthropometry between various cultural backgrounds and races. In this sense, the perception of the female shape varies between European and Eastern countries.^9–11^

To our knowledge, there is no study on the breast measurements of Saudi women. The aim of this study was to identify the descriptive measurements of the breast in the sample population of young nulliparous Saudi women and to determine the mean breast parameters and natural anatomical proportions of the studied population. By identifying breast morphometry from the values obtained in this study, we aimed to produce a reference range of average breast values, as it will benefit patients undergoing mammoplasty and reconstructive breast surgery. These data are essential to achieve the best possible aesthetic outcomes and for comparison with results obtained from women of other racial backgrounds.

## MATERIALS AND METHODS

### Subject Recruitment

Fifty-four nulliparous Saudi women of 20–25 years old were enrolled from patients attending the hand Surgery clinic and from medical student volunteers. The inclusion criteria were good healthy nulliparous female with normal physical development and body mass index (BMI) of 20–25 kg/m^2^. All potential subjects with chest deformity, previous surgery, and endocrine, or general health issues that may affect the measurements, were excluded. All recruited subjects signed written informed consent forms following approval from the research committee of Care National Hospital.

### Research Parameters

**Fig. 1.**
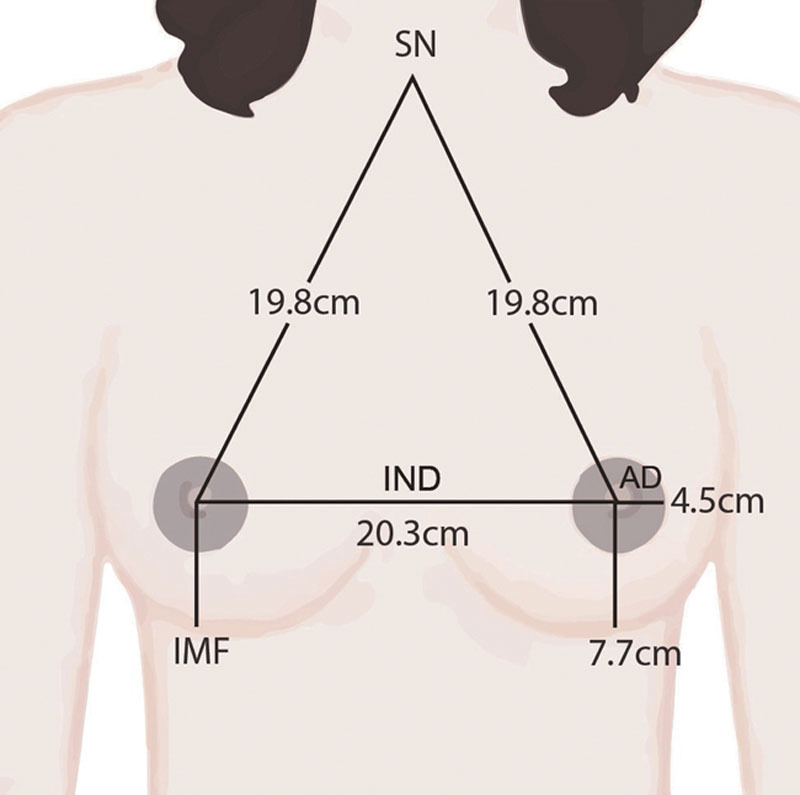
Breast parameters. SN, sternal notch; IMF, inframammary fold; IND, internipple distance; and AD, areolar diameter.

The general parameters measured were age, weight, height, and BMI. The BMI was calculated using the following formula: BMI = weight/height^2^ (kg/m^2^). Breast parameters such as the sternal notch-nipple length (SN), the internipple distance (IND), the distance from the nipple to the inframammary fold (IMF), the distance from the lower edge of the areola to the IMF, and the areolar diameter (AD) were measured (Fig. 1). The measurements were taken by a tape measure in a warm room with subjects in erect position with arms placed by their sides. To increase data reproducibility, all the measurements were taken by 1 investigator and checked by the other investigator on 2 occasions. The average of these measurements was taken for each subject.

### Data Analysis

All data are represented by the mean ± SD. For comparison, statistical analysis was performed using the Student’s *t* test. Pearson regression values were used for correlation. All statistical analyses were performed using the program Prism Graph Pad (GraphPad Software Inc., San Diego, Calif.).

## RESULTS

In this study, 54 young women volunteered for breast measurements. The mean age, BMI, height, and weight of the subjects were 22.1 ± 1.2 years, 21.8 ± 3.1 kg/m^2^, 162.1 ± 5 cm, and 57.4 ± 8.6 kg, respectively (Table 1). The mean values of the measured breast parameters were 19.8, 20.3, 7.7, 5.5, and 4.5 cm for SN, IND, distance from nipple to IMF, distance from the lower edge of areola to the IMF, and AD, respectively (Table 1).

**Table 1.** Physical Characteristics and Breast Measurements (N = 53)

	Mean	SD
Age, y	22.1	1.2
Height, cm	162.1	5.0
Weight, kg	57.4	8.6
BMI, kg/m^2^	21.8	3.1
SN, cm	19.8	2.5
IND, cm	20.3	2.3
Nipple to IMF, cm	7.7	1.6
Lower end of areola to IMF, cm	5.4	1.4
AD, cm	4.5	1.4

There were no statistically significant differences in most parameters between the left and right breasts. However, there were statistically significant differences in the distance from the edge of the areola to the IMF between the left and right breasts, with values of the left breast higher than those of the right breast (*P* < 0.05, n = 54). There were also no statistically significant differences found in the distance from the nipple to the IMF between the left and right breasts as shown in (Table 2; *P* = 0.06).

**Table 2.** Comparison of the Measurements of the Right and Left Breasts (N = 53)

	Right Breast (Mean ± SD), cm	Left Breast (Mean ± SD), cm	*P*
SN	19.8 ± 2.5	19.8 ± 2.5	0.89
Nipple to IMF	7.6 ± 1.6	7.8 ± 1.6	0.06
End of areola to IMF	5.4 ± 1.4	5.5 ± 1.5	<0.05
AD	4.5 ± 1.4	4.5 ± 1.4	0.93

We investigated for any correlations between patient characteristics such as age, height, weight, and BMI. We found a positive correlation between weight and BMI and between SN, IND, distance from nipple to IMF, and AD. In contrast, no correlation was found between the age and height of subjects. The breast measurements, Pearson’s correlation coefficients, and *P* values are shown in Table 3.

**Table 3.** Correlation of Patients Characteristics and Breast Measurements (N = 53)

	SN		IND		Nipple to IMF		End of Areola to IMF		AD	
	Pearson *r*	*P*	Pearson *r*	*P*	Pearson *r*	*P*	Pearson *r*	*P*	Pearson *r*	*P*
Age	−0.13	0.17	0.19	0.16	0.07	0.43	0.04	0.67	0.18	0.06
Height	0.04	0.70	0.20	0.15	0.20	<0.05	0.19	0.06	-0.05	0.58
Weight	0.63	<0.0001	0.39	<0.005	0.33	<0.05	0.16	0.26	0.28	<0.05
BMI	0.63	<0.0001	0.32	<0.05	0.29	<0.005	0.08	0.41	0.30	<0.005

## DISCUSSION

This study evaluated the mean breast parameters in a sample of young nulliparous Saudi women to establish reference values for the general Saudi female population. Proportion is an important aspect of aesthetics, and to get the best outcome in breast surgeries, some standards must be maintained. These standards may be regarding protocoled measurements of the breast or comparison with an average breast in a given population.

Numerous earlier studies have attempted to determine ideal breast parameters or mean breast anthropometry, and some of them used models.^12–14^ Increased interest in the calculation of mean breast parameters in certain populations has been noted in most recent studies, probably because of increased patient demand for breast surgeries.^15^ Moreover, a review published in 2014 suggested that breast size may be a risk factor for developing breast cancer.^16^ Hence, calculating mean breast parameters in a population seems to have become highly important, even though results were not sharply conclusive. However, evidence indicated that patients with large breasts are at higher risk of breast cancer, and the individuals who underwent breast augmentation surgery have a lower risk of breast cancer compared with the general population.^16^

Noteworthy differences and similarities were found between our measurements and those of subjects of other nationalities and races. For instance, findings of a study in China that measured breast parameters of 125 Chinese women revealed small but important differences compared with Saudi women. Chinese women of a similar age range as the Saudi women in our study had a mean AD of 3.32 ± 0.35 cm compared with a mean AD 4.5 ± 1.4 cm in Saudi women as indicated in our results, which is a difference of about 1 cm (Table 4).^17^ Furthermore, a comparable difference of about 1 cm in mean AD was found when our results were compared with those of a Turkish population of a similar age range with a mean AD of 3.6 ± 0.9 cm (Table 4).^4^ Another study conducted in the United Kingdom showed the mean AD to be about 50 ± 19 mm, with a 0.50 cm difference from our findings, although the United Kingdom women studied were of a wider age range (15–88 years) and had a mean BMI of 24 kg/m^2^, which explains the difference (Table 4). In general, the increased AD could be associated with increased weight. Our analysis showed a significant positive correlation between weight and AD, confirming the observed difference between the Saudi and United Kingdom sample populations.^18^ Studies in relevant literature, to our knowledge, showed no statistically significant differences in AD between the left and right breasts, which is consistent with our findings.

**Table 4.** Comparative Analysis Between Breast Measurements of Kingdom of Saudi Arabia Population and Different Documented Populations (N = 53)

	Current Findings Mean	Other Populations	P*	Reference
AD	4.5	Chinese 3.32	<0.0001	16
		Turkish 3.60	<0.0001	4
		British 5.00	<0.001	17
SN	19.8	Chinese 19.05	<0.01	16
		Turkish 19.6	0.16	4

* *P* using ANOVA.

The mean SN in our study was 19.8 cm, which is slightly higher than that of the Chinese women (19.05 cm) but close to that of the Turkish women (19.6 cm; Table 4). The mean IND values in most published articles were found to be similar to that of our study.^4,17^

In the daily practice of plastic surgery, it is especially important to use the same anthropometric tool. The same device or instrument should be used pre-, peri-, and postoperatively to reduce the probability of differences in measurements. A study that compared results of breast parameters measured using a tape measure and a compass revealed significant differences in results between the 2 methods of measurement used.^19^ Limitations of our study include its sample size and further study in a larger population will be beneficial. Moreover, additional parameter needed to be measured to obtain average breast volume, shape, and surface area. Finally, the age group and BMI ranges in our study were very narrow. Hence, it is important to note that the current results may not apply to Saudi women who are not from this age or weight groups. Otherwise, forcing these parameters in an obese patient may result in a disappropriate breast for her size. Hence, our study should be considered as the baseline reference values for young nulliparous nonobese Saudi women. We plan to conduct further studies to integrate this in the future.

In conclusion, this anthropometric study of young Saudi women is the first for this population. The aim of this study was to measure the descriptive indices of the breast, to determine the average values of breast parameters, in young Saudi females. We reckon that it will be useful for surgeons who perform breast reconstruction, augmentation, and reduction. Furthermore, from the viewpoint of patients, surgeons stand to avoid unfavorable surgical outcomes if parametric measures are taken objectively and discussed before aesthetic surgery.
